# Comparative evaluation of chlorhexidine with or without hyaluronic acid on early periodontal wound healing

**DOI:** 10.6026/973206300223747

**Published:** 2026-06-30

**Authors:** Abhima Kumar, Kousain Sehar, Sabiha Parwaiz, Mohd Irfan, Chahat Puri, Danish Hamid

**Affiliations:** 1 Department of Periodontology and Oral Implantology, Indira Gandhi Government Dental College & Hospital, Jammu, Jammu and Kashmir, India

**Keywords:** Gingivectomy, chlorhexidine, hsyaluronic acid

## Abstract

Achieving uncomplicated tissue recovery remains a major challenge after periodontal surgery. Therefore, it is of interest to evaluate the 10 gingivectomy patients presenting with gingival enlargement. Post-operatively, participants used either a combined 0.2% CHX+HA rinse or CHX alone. The CHX+HA cohort exhibited significantly superior early-stage wound healing outcomes. Thus, data shows combining chlorhexidine with hyaluronic acid enhances early periodontal tissue healing.

## Background:

This minor oral surgery involves resecting part of the gingiva to minimize the soft tissue wall enclosing a periodontal pocket [1]. Chlorhexidine (CHX), regarded as the gold standard in chemical plaque control, is widely used as a mouthrinse [2, 3]. Chlorhexidine has been demonstrated to reduce dental plaque accumulation and gingival inflammation [4] Evidence from systematic reviews indicates that post-operative use of chlorhexidine can improve wound healing and decrease the incidence of complications [5, 6]. Hyaluronan in hyaluronic acid promotes efficient wound healing by stimulating cell migration, fibroblast proliferation [7]. Also it exhibits bacteriostatic, proangiogenic and anti-inflammatory properties [8]. Therefore it is of interest to assess the biochemical and clinical results of chlorhexidine mouthwash alone and chlorhexidine in combination with hyaluronic acid.

## Methods:

After approval by the Institutional Ethical Committee participants were selected among patients seeking care at the Postgraduate Department of Periodontology & Oral Implantology, Indira Gandhi Govt. Dental College & Hospital, Jammu, Jammu and Kashmir. For this double-blinded, split-mouth RCT, ten patients were selected, aging 18-60 years (4 men and 6 women). Systemically healthy patients with gingival enlargement in which bottom of pockets is not apical to mucogingival junction are included in the study. On the other hand, patients with history of antimicrobial use in the last six months, pregnancy and history of tobacco use were excluded from the study. After selecting the study subjects, the sites were divided into:

[1] Group-A: Gingivectomy followed by 0.2% chlorhexidine mouthwash with Hyaluronic acid.

[2] Group-B: Gingivectomy followed by 0.2% chlorhexidine mouthwash alone.

## Surgical procedure:

An informed written consent was taken from the patient before participation in the study. After meticulous completion of Phase I therapy, a gingivectomy procedure was performed on all 10 patients. Post-operative instructions were given and mouth rinse was prescribed as per the allocated group. Patient was recalled after 24 hrs and 1 week for evaluation and parameters were recorded.

## Results:

Descriptive statistics were expressed as mean ± standard deviation (SD) for all continuous variables, including plaque index, sulcular bleeding index, visual analog scale (VAS), interleukin-6 (IL-6) levels and wound healing index (Table 1). The mean pre-operative plaque index value observed in group A was 0.53 ± 0.04 and 0.59 ± 0.05 in Group B (Figure 1 - see PDF). The mean sulcular bleeding index was 0.32 ± 0.05 in Group A and 0.37 ± 0.05 in Group B (Figure 2 - see PDF). After applying paired t-test, at 24 hours post-operatively, the mean VAS score was found to be 3.5 ± 0.4 in Group A which was reduced to 2.0 ± 0.2 on 7th day and for group B, a value of 4.0 ± 0.5 was reduced to 2.7 ± 0.3 on 7th day (Figure 3 - see PDF). A reduction in pain was demonstrated in both groups on 7th day, with Group A showing comparatively lower scores; however, the differences between the groups were not statistically significant (p > 0.05). Paired t-test was applied after calculating the mean values. At 24 hrs post- operatively, the mean IL-6 level value was observed to be 10.5 ± 0.4 pg/mL in Group A and 10.9 ± 0.5 pg/mL in Group B respectively. However, on 7th day, the mean IL-6 level decreased to 9.45 ± 0.28 pg/mL in Group A and 10.57 ± 0.33 pg/mL in Group B respectively (Figure 4 - see PDF). However, the intergroup differences were not statistically significant (p > 0.05). For Early Wound Healing Index (EHI) after applying unpaired t-test, at 7 days post-operatively, the mean wound healing index was found to be 4.32 ± 0.08 in Group A and 3.85 ± 0.11 in Group B respectively. Although Group A demonstrated higher healing scores, the difference between the groups was not statistically significant (p > 0.05) (Figure 5 - see PDF).

## Discussion:

During healing by secondary intention, as seen in gingivectomy procedures, wounds can be associated with discomfort and delayed healing compared to healing by primary intention. To enhance healing outcomes and minimise complications, various adjunctive modalities have been introduced. The present comparative evaluation of chemical agents highlights the role of chlorhexidine (CHX), with or without hyaluronic acid (HA), in modulating early periodontal wound healing. Chlorhexidine has long been regarded as the gold standard antiplaque agent due to its broad-spectrum antimicrobial activity and substantivity. Its effectiveness in reducing plaque accumulation by 30-80% makes it particularly valuable in the immediate post-operative period, when mechanical plaque control is compromised [9]. The findings of this study are consistent with Kozlovsky *et al.* [10] and Lang *et al.* [11] who demonstrated improved early wound healing and reduced plaque accumulation following CHX use in post-operative settings. Graziani *et al.* (2024) reported that CHX-based rinses significantly improved early wound healing, as evidenced by lower Early Healing Index (EHI) scores and reduced gingival inflammation compared to untreated controls [9]. Coelho *et al.* (2020) reported that CHX mouthwash induces oxidative stress and necrotic cell death in fibroblasts even at concentrations below those used clinically [12]. Hyaluronic acid or Hyaluronan, a linear polysaccharide found natively in the synovial fluid and the extracellular matrix of connective tissue, has emerged as a promising adjuvant in periodontal therapy. Owing to the inherent bacteriostatic, anti-inflammatory, anti-oedematous, osteo-inductive and pro-angiogenetic qualities, HA has been said to have several positive effects on wound healing [13, 14]. In Group A VAS showed lower score compared to group B which could be attributed to its hydration and film forming abilities Kozlovsky *et al* [10]. A comparative *in vitro* study by Coelho *et al.* [12] demonstrated that while chlorhexidine displayed higher cytotoxic effects on human gingival fibroblasts, hyaluronic acid retains better cell viability and supports fibroblast migration, thereby enhancing the wound healing environment. It suggests that HA can mitigate the biological damage associated with chlorhexidine exposure, although it does not eliminate its cytotoxic effects. The synergistic effect of combining CHX with HA is of particular interest. In the randomised clinical trial by Graziani *et al.* (2024) [9] the combination of Hyaluronic acid with chlorhexidine resulted in improved healing outcomes compared to CHX alone, especially during the early post-operative period (3-7 days). Patients treated with CHX + HA demonstrated significantly improved soft tissue closure, reduced dehiscence and better control of inflammation. These findings suggest that while CHX primarily addresses the microbial component of wound healing, HA contributes to the biological enhancement of tissue repair. Interleukin-6 (IL-6) is a multifunctional pro-inflammatory cytokine that plays a central role in the early phase of wound healing, acting as a key mediator linking acute inflammation to tissue repair. IL-6 and IL-3 act in a synergistic way to increase hematopoietic stem cell proliferation *in vitro*, where IL-6 activates cells at the G0 stage to enter the G1 phase [15, 16]. Following periodontal surgical procedures such as gingivectomy, IL-6 levels in gingival crevicular fluid and serum rise rapidly within the first 24 hours, reflecting the immediate host inflammatory response and typically peak between 48-72 hours before gradually declining as healing progresses toward the proliferative phase and return to baseline within 7-14 days [17, 18, 19]. This transient elevation is associated with neutrophil recruitment, activation of acute-phase protein synthesis and initiation of fibroblast-mediated tissue repair. HA protects the oral mucosa and it retains the active molecule at the site of action to prolong their effect. It also seems to be effective in reducing the inflammatory response if used as an adjunct to non-surgical and surgical periodontal therapies demonstrated through clinical parameters [20, 21]. Accordingly, CHX combined with HA can be hypothesized to provide a synergistic effect by maintaining antimicrobial action while HA supports faster resolution of inflammation and its mediators. In the current study, the combination of CHX+HA exhibited a positive impact on wound healing showing higher mean value healing scores on day 7, although the difference was not statistically significant. There was a greater reduction in VAS scores in the CHX+HA group, but the authors failed to find significant intergroup differences in it. More trials inclusive of a larger group with extended follow-up periods to evaluate the long-term benefits of CHX and HA combination therapies are indicated.

## Conclusion:

Chlorhexidine with Hyaluronic acid and Chlorhexidine alone are effective in enhancing the healing process, with CHX+HA exhibiting slightly better healing outcomes. Chlorhexidine is an important component of post-operative care; the incorporation of biologically active agents such as hyaluronic acid acts synergistically in further enhancing the healing cascade.

## Source of funding:

Nil

## Figures and Tables

**Table 1:** Comparative evaluation of clinical and biochemical parameters between Group A and Group B

**Parameter**	**Time**	**Group A (Mean ± SD)**	**Group B (Mean ± SD)**	**Inference(comparison Group A & Group B)**
Plaque Index	Pre-operative	0.53 ± 0.04	0.59 ± 0.05	Baseline
Sulcular Bleeding Index	Pre-operative	0.32 ± 0.05	0.37 ± 0.05	Baseline
VAS	24 hours	3.5 ± 0.4	4.0 ± 0.5	Not significant
VAS	Day 7	2.0 ± 0.2	2.7 ± 0.3	Not significant
IL-6	24 hours	10.5 ± 0.4	10.9 ± 0.5	Not significant
IL-6	Day 7	9.45 ± 0.28	10.57 ± 0.33	Not significant
Wound Healing Index	Day 7	4.32 ± 0.08	3.85 ± 0.11	Not significant
Group A: Chlorhexidine + Hyaluronic acid
Group B: Chlorhexidine alone

## References

[1] Pai DD (2023). J Oral Health Comm Dent.

[2] Addy M, Moran JM. (1997). Periodontol 2000.

[3] Löe H, Schiott CR. (1970). J Periodontal Res..

[4] Jones CG (1997). Periodontol 2000.

[5] Romero-Olid MN (2023). Antibiotics (Basel)..

[6] Casarin M (2023). Eur J Oral Sci..

[7] Casarin M (2023). Front Oral Health..

[8] Chen WY, Abatangelo G. (1999). Wound Repair Regen..

[9] Graziani F (2024). Clin Oral Investig..

[10] Kozlovsky A (2007). J Clin Periodontol..

[11] Lang NP (1973). J Periodontol..

[12] Coelho AS (2020). Odontology..

[13] Shukla K (2024). Cureus.

[14] Fu SW (2026). BMC Oral Health..

[15] Kishimoto T (1989). Blood..

[16] Marín Fermín T (2024). Journal of ISAKOS..

[17] Loos BG (2005). J Periodontol..

[18] Graves DT, Cochran D. (2003). destruction. J Periodontol..

[19] Nishimoto N (2004). Arthritis Rheum..

[20] Mallineni T (2020). J Dent Res Dent Clin Dent Prospects..

[21] Chandra N (2026). Bioinformation..

